# Telehealth Treatment of Patients with Bipolar Depression during the COVID-19 Pandemic: Comparative Safety, Patient Satisfaction, and Effectiveness to Prepandemic In-person Treatment

**DOI:** 10.1192/j.eurpsy.2024.470

**Published:** 2024-08-27

**Authors:** M. Zimmerman

**Affiliations:** Psychiatry, Brown University, Providence, United States

## Abstract

**Introduction:**

The COVID-19 pandemic prompted a transition from in-person to telehealth psychiatric treatment. There are no studies of partial hospital telehealth treatment for bipolar disorder.

**Objectives:**

In the present report from the Rhode Island Methods to Improve Diagnostic Assessment and Services (MIDAS) project, we compared the effectiveness of partial hospital treatment of patients with bipolar depression treated virtually versus in-person.

**Methods:**

Outcome was compared in 76 patients with bipolar depression who were treated virtually from April, 2020 to December, 2022 to 130 patients who were treated from May, 2017 to January 2020. The patients completed self-administered measures of patient satisfaction, symptoms, coping ability, functioning, and general well-being.

**Results:**

In both the in-person and telehealth groups patients with bipolar depression were highly satisfied with treatment and reported a significant reduction in symptoms from admission to discharge. Both groups also reported a significant improvement in positive mental health, general well-being, coping ability, and functioning. Suicidal ideation was reduced in both groups. No patients attempted suicide. A large effect size of treatment was found in both treatment groups. The length of stay and the likelihood of staying in treatment until completion were significantly greater in the virtually treated patients.

**Conclusions:**

Telehealth delivery of partial hospital level of care for patients with bipolar depression was as safe and effective as in-person treatment.

**Disclosure of Interest:**

None Declared

